# Learning and Memory Deficits in Male Adult Mice Treated with a Benzodiazepine Sleep-Inducing Drug during the Juvenile Period

**DOI:** 10.3389/fnins.2016.00339

**Published:** 2016-07-20

**Authors:** Yusuke Furukawa, Kentaro Tanemura, Katsuhide Igarashi, Maky Ideta-Otsuka, Ken-Ichi Aisaki, Satoshi Kitajima, Masanobu Kitagawa, Jun Kanno

**Affiliations:** ^1^Division of Cellular and Molecular Toxicology, Biological Safety Research Center, National Institute of Health SciencesTokyo, Japan; ^2^Department of Comprehensive Pathology, Graduate School, Tokyo Medical and Dental UniversityTokyo, Japan; ^3^Laboratory of Animal Reproduction and Development, Graduate School of Agricultural Science, Tohoku UniversitySendai, Japan; ^4^Life Science Tokyo Advanced Research Center, Hoshi University School of Pharmacy and Pharmaceutical SciencesTokyo, Japan; ^5^Japan Bioassay Research Center, Japan Organization of Occupational Health and SafetyHadano, Japan

**Keywords:** sleep-inducing drug, triazolam, zolpidem, GABA receptor signal, behavioral battery test

## Abstract

Gamma-aminobutyric acid (GABA), the major inhibitory neurotransmitter in the mammalian central nervous system, is also known to be important for brain development. Therefore, disturbances of GABA receptor (GABA-R) mediated signaling (GABA-R signal) during brain development may influence normal brain maturation and cause late-onset brain malfunctions. In this study, we examined whether the stimulation of the GABA-R signal during brain development induces late-onset adverse effects on the brain in adult male mice. To stimulate the GABA-R signal, we used either the benzodiazepine sleep-inducing drug triazolam (TZ) or the non-benzodiazepine drug zolpidem (ZP). We detected learning and memory deficits in mice treated with TZ during the juvenile period, as seen in the fear conditioning test. On the other hand, ZP administration during the juvenile period had little effect. In addition, decreased protein expression of GluR1 and GluR4, which are excitatory neurotransmitter receptors, was detected in the hippocampi of mice treated with TZ during the juvenile period. We measured mRNA expression of the immediate early genes (IEGs), which are neuronal activity markers, in the hippocampus shortly after the administration of TZ or ZP to juvenile mice. Decreased IEG expression was detected in mice with juvenile TZ administration, but not in mice with juvenile ZP administration. Our findings demonstrate that TZ administration during the juvenile period can induce irreversible learning and memory deficits in adult mice. It may need to take an extra care for the prescription of benzodiazepine sleep-inducing drugs to juveniles because it might cause learning and memory deficits.

## Introduction

Normal brain development requires various neuronal signals must be activated at the appropriate timing and with the proper extent in the developmental brain (Rice and Barone, 2000). Excitatory glutamate receptor (Glu-R) signals and inhibitory gamma-aminobutyric acid (GABA) receptor (GABA-R) signals are the most important neuronal signals in the adult brain. These neuronal signals are also known to be important for brain development. These signals have roles in neuronal cellular proliferation and differentiation, neuronal migration, the construction of neuronal circuits, and the reorganization of neuronal circuits (Luján et al., 2005). Therefore, the stimulation of these neuronal signals with external factors, such as chemical compounds, may interfere with normal brain development and result in late-onset functional deficits during adulthood. We have previously reported that the transient activation of Glu-R signals in the prenatal mouse brain with domoic acid results in aberrant emotional behavior, as well as learning and memory deficits, as revealed by a mouse behavioral battery tests (BBT) (Tanemura et al., 2009). On the other hand, Haas et al. have shown that prenatal GABA-R signal activation with the anxiolytic drug Diazepam (DZP) leads to the inhibition of neuronal migration and the disruption of cerebral cortex neuronal circuits (Haas et al., 2013). In addition, Shen et al. have shown that neonatal activation of GABA-R signals by DZP results in increased anxiety-like behavior (Shen et al., 2012). However, the behavioral tests applied in these studies were limited, and their results were not analyzed in an integrated manner. We therefore believe that analyses of several behavioral tests during the adult stage following the activation of GABA-R signals during brain development are critically needed.

In this study, we report the results of the BBT that we conducted. These include the open field test, the light/dark transition test, the elevated plus maze test, the contextual/cued fear conditioning test, and the pre-pulse inhibition test. The results of these tests will help us to understand the effects of GABA-R signal activation during brain development with the benzodiazepine (BZD) sleep-inducing drug triazolam (TZ: original brand name “Halcion”) or the non-BZD drug zolpidem (ZP: originally marketed as “Ambien” and available worldwide under many brand names) on behavior during the adult stage (Pakes et al., 1981; Holm and Goa, 2000). These sleep-inducing drugs have similar pharmacokinetic and pharmacodynamic effects in humans (Lobo and Greene, 1997). They are preferentially used as drugs for the treatment of insomnia owing to their lack of carryover effects on the next day (Neubauer, 2007). Their reported side effects include drug-dependence, withdrawal symptoms, psychiatric symptoms (excitement stimulation, confusion, aggression, noctambulation, hallucinations, delusions, and agitation), transient anterograde amnesia before and after sleeping, or arousal during sleep (Pakes et al., 1981; Jonas et al., 1992; Toner et al., 2000; Greenblatt and Roth, 2012). Treatment of sleep disorders in children using hypnotic drugs is common (Kahn et al., 1989; Stores, 1996; Owens et al., 2003; Weiss and Garbutt, 2010; Felt and Chervin, 2014), although their safety in children has not been established (FDA, 2008, 2013). These chemicals act by suppressing excitatory neuronal activity by inducing hyperpolarization following the cellular influx of chloride ions when they bind to GABA (A)-R alpha and gamma receptors. GABA (A)-R is found as pentamers of a combination of 19 subunits (α1–6, β1–3, γ1–3, δ, ϵ, θ, π, and ρ1–3). The BZD chemical TZ binds to GABA (A)–R α1, 2, 3, 5, and 6 subtypes in any combination, along with the γ2 subtype. The non-BZD chemical ZP binds only to combinations of GABA (A)–R α1 and γ2 subtypes (Rudolph and Knoflach, 2011).

## Materials and methods

### Animal experiment

**Figure 1:**
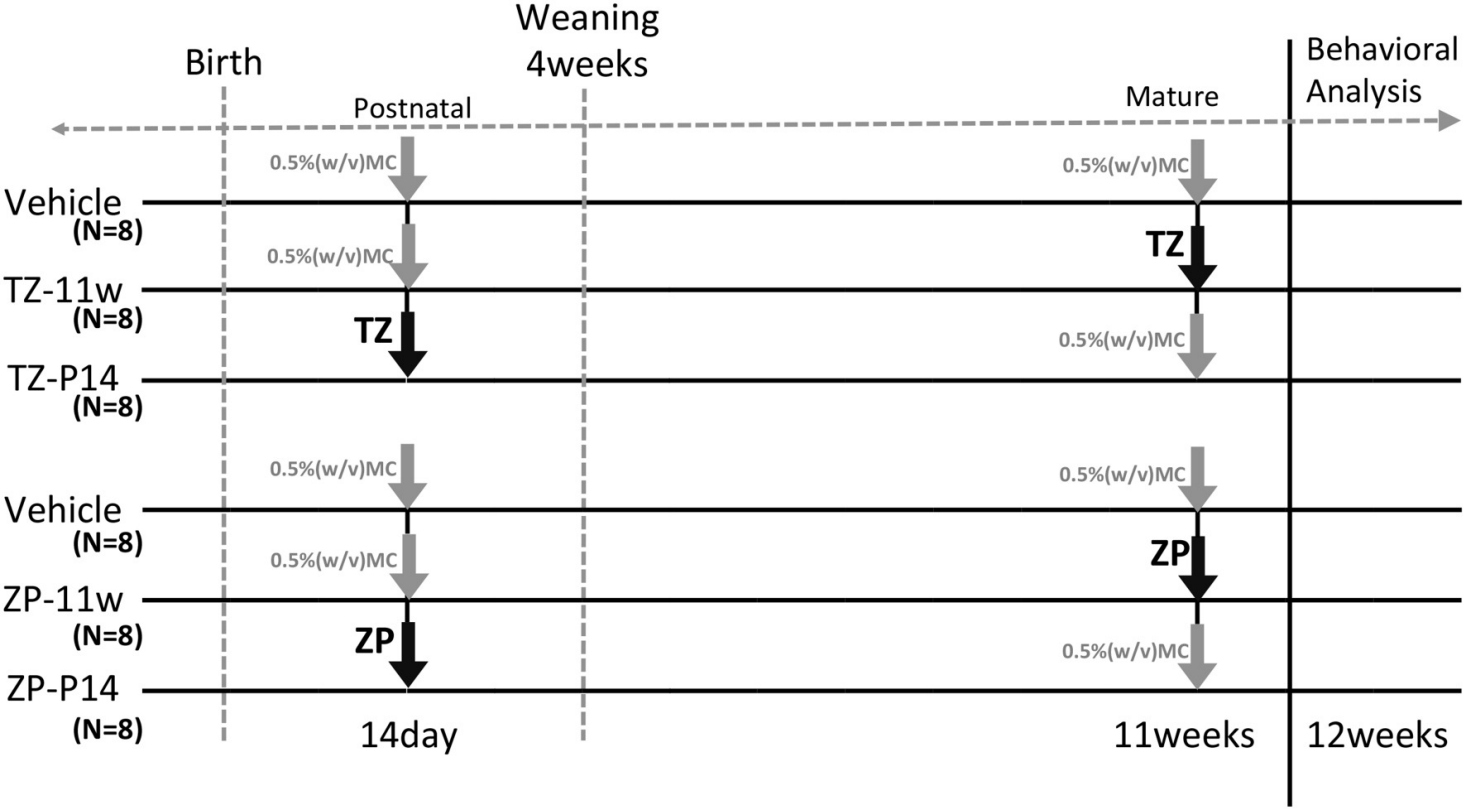
**Experimental schedule**. This figure describes the experimental schedules for triazolam (TZ) and zolpidem (ZP) treatment. The gray arrow indicates vehicle and the black arrows indicate TZ 1 mg/kg or ZP 50 mg/kg. All mice received the drug three times by gavage. The two ages of 2 weeks (2 w) and 11 weeks (11 w) were selected. TZ and ZP were administered to the mice at one of the two time points and vehicle was administered at the other two time points. The mice were weaned at 4 weeks. Male mice were selected and were housed in new cages (4 mice per cage). A behavioral battery tests (BBT) was carried out starting at 12 weeks of age. Each group consisted of eight mice (6 group, 48 mice total).

All animal experiments were conducted with permission from the Animal Ethics Committee at the National Institute of Health Sciences. Pregnant female C57BL/6NCrSlc mice at embryonic day 11 were purchased from Japan SLC Inc. (Shizuoka, Japan). The mice were housed in plastic cages and maintained under a 12-h light/12-h dark cycle with water and chow (CRF-1, Oriental Yeast Co. Ltd., Tokyo, Japan) provided *ad libitum*. Triazolam (TZ: 8-chloro-6-(2-chlorophhenyl)-1-methyl-4H-1, 2, 4 -triazolo[4,3-a]-1, 4-benzodiazepine; Sigma Aldrich Co. Steinheim, Germany), and Zolpidem (ZP: N,N,6-Trimethyl-2-(4-methylphenyl)-imidazo[1,2-a]pyridine-3-acetamide; Sigma Aldrich Co., Steinheim, Germany) were dissolved in 0.5% (w/v) methyl cellulose solution (Wako Pure Chemical Industries, Ltd. Osaka, Japan) and administered by gavage at the doses of 1 and 50 mg/kg. In the vehicle group, 0.5% (w/v) methyl cellulose solution (MC) was administered by gavage. Administration and experimental schedules are shown in Figure 1. Mice in the Vehicle group (8 mice per experimental group, 16 mice total) were treated with 0.5% (w/v) MC at 2 weeks and 11 weeks of age. The TZ-2w group (8 mice) was treated with TZ at 2 weeks of age and with 0.5% (w/v) MC at 11 weeks of age. The ZP-2w group (8 mice) was treated with ZP at 2 weeks of age and with 0.5% (w/v) MC at 11 weeks of age. The TZ-11w group (8 mice) was treated with 0.5% (w/v) MC at 2 weeks of age and with TZ at 11 weeks of age. The ZP-11w group (8 mice) was treated with 0.5% (w/v) MC at 2 weeks of age and with ZP at 11 weeks of age. Subsequently, a series of BBT were conducted at 12 weeks of age. After the BBT, the brains of the mice were dissected, and the hippocampus was removed for biochemical analysis at 13 weeks of age.

### Mouse behavioral battery test

We conducted a behavioral battery test (BBT), including the open field test (OF), the light/dark transition test (LD), the elevated plus maze test (EP), the contextual/cued fear conditioning test (FZ), and the additional pre-pulse inhibition test (PPI). For the OF, LD, EP, and FZ methods details, refer to the Tanemura et al. (2009). In the present study, we added the PPI further for information processing analysis. Experimental apparatuses and image analysis software was obtained from O'Hara & Co., Ltd., Japan. Image analysis software (Image OF4, Image LD2, Image EP2, and Image FZ2) were developed using the public domain ImageJ program. All experiments were performed with 8 mice per group (TZ experimental: 3 groups, ZP experimental: 3 groups). We thus had 6 groups for a total of 48 mice. The experimental tests were conducted between 13:30 and 16:30. The level of background noise during BBT was about 50 dB. After each trial, the apparatus was wiped and cleaned. The pre-pulse inhibition test apparatus consists of a light source and a sound system, and a startle measurement load cell. These are set into a soundproof box. The software for the operation of the apparatus and the data analysis is the SR-9040 (O'Hara & Co., Ltd., Tokyo, Japan). The white background noise level is set to 70 dB in the soundproof box. The mouse is put into a plastic cylinder and kept there for 90 s before the test. The test schedule consists of three blocks, and the total trial time is 30 min. Breakdown of each block is as follows: 80, 85, 95, 100, 105, and 110 dB pulse × 3 (acclimation block), 120 dB pulse × 10 (acoustic startle response block). The combinations of pre-pulse are 80–120, 85–120, 95–120, 100–120, 105–120 dB, with a delay of 100 ms × 6 (pre-pulse inhibition measurement block). These combinations were presented in a pseudorandom order, such that each trial type was presented once within a block. The inhibition ratio (%) of the startle response is calculated as follows: (1–pre-pulse [80, 85, 95, 100, or 105 dB] startle response value / acoustic startle response value) × 100.

### Western blotting

Hippocampal extracts were dissolved in Tris-buffered saline (pH 7.4) containing protease inhibitors (Nacalai Tesque, Inc., Kyoto, Japan) and phosphatase inhibitors (Nacalai Tesque, Inc., Kyoto, Japan). Equal volume of the total protein solutions were added to 2 × sample buffer solution (Nacalai Tesque, Inc., Kyoto, Japan). The amount of protein was quantified using Qubit protein assay kits (Life Technologies Co., California, USA). The protein samples (30 μg/well) were subjected to SDS-PAGE (7.5% polyacrylamide), and transferred to a nitrocellulose membrane. The membranes were blocked in Blocking-one (Nacalai Tesque, Inc., Kyoto, Japan) at room temperature for 90 min and incubated with primary antibodies, such as those against acetyl-tubulin (sc-23950, Santa Cruz Biotechnology, Inc., California, USA), MAP2 (sc-20172), GluR1 (T9026, Sigma Aldrich Co., Steinheim, Germany), and GluR4 (SAB450126) overnight at room temperature. The membranes were then washed with phosphate buffered saline (pH 7.4) with 0.05% Tween-20 (PBS-T). The membranes were then incubated with peroxidase-conjugated secondary antibodies for 2 h at room temperature. After several PBS-T washes, the membranes were incubated with chemi-lumi one L (Nacalai Tesque, Inc., Kyoto, Japan) for signal production. The signal was photographed using an LAS-3000 (Fujifilm Co., Ltd., Tokyo, Japan). The intensity of each protein band was measured using the Gel Analyzer program in ImageJ, and normalized to the intensity of the acetyl-tubulin band using the following formula: (intensity of each protein band/acetyl-tubulin band intensity) × 100. We present each normalized band intensity as a percentage of its intensity in the Vehicle group. The changes in protein expression associated with TZ and ZP treatment were calculated as follows: Ratio (%) = (Vehicle, TZ and ZP group individual value/Vehicle group average value) × 100.

### Quantitative RT-PCR

Duplicate homogenate hippocampal samples were treated with DNaseI (amplification grade, Invitrogen Corp., Carlsbad, CA, USA) for 15 min at room temperature. They were then incubated with Super-Script II (Invitrogen) for 50 min at 42°C for reverse transcription. Quantitative real time PCR was performed using an ABI PRISM 7900 HT sequence detection system (Applied Biosystems, Foster City, CA, USA) using SYBR Premix Ex Taq (Takara Bio Inc., Japan) with initial denaturation at 95°C for 10 s followed by 45 cycles of 5 s at 95°C and 60 s at 60°C. Ct values were obtained. The primers were synthesized by FASMAC Co., Ltd., Japan. Expression of genes of interest was normalized to that of *Actb* and presented as fold change over baseline using the delta-delta CT method. Fold changes of relative gene expression levels compared to those of Vehicle animals were calculated (Livak and Schmittgen, 2001).

Primers

*Arc*: forward 5′-TACCGTTAGCCCCTATGCCATC-3′, reverse 5′-TGATATTGCTGAGCCTCAACTG-3′

*c-Fos*: forward 5′-ATGGGCTCTCCTGTCAACACAC-3′, reverse 5′-ATGGCTGTCACCGTGGGGATAAAG-3′

*Nr4a1*: forward 5′- TTAAGAGGTGGGTCGGGTTC -3′, reverse 5′- GCAATCCTTCTCGCACACTA -3′

*ActB*: forward 5′-GGACTCATCGTACTCCTGCTT-3′, reverse 5′-GAGATTACTGCTCTGGCTCCT-3′.

### Statistical analysis

Statistical analysis was conducted using Prism 5.04 (SAS Institute, California, USA). Data was analyzed using Student's unpaired *t*-tests. Values in graphs are expressed as the mean ± standard error of the mean (SEM).

## Result

### Results of behavioral battery tests in the TZ-11w, TZ-2w, ZP-11w, and ZP-2w groups

**Figure 2:**
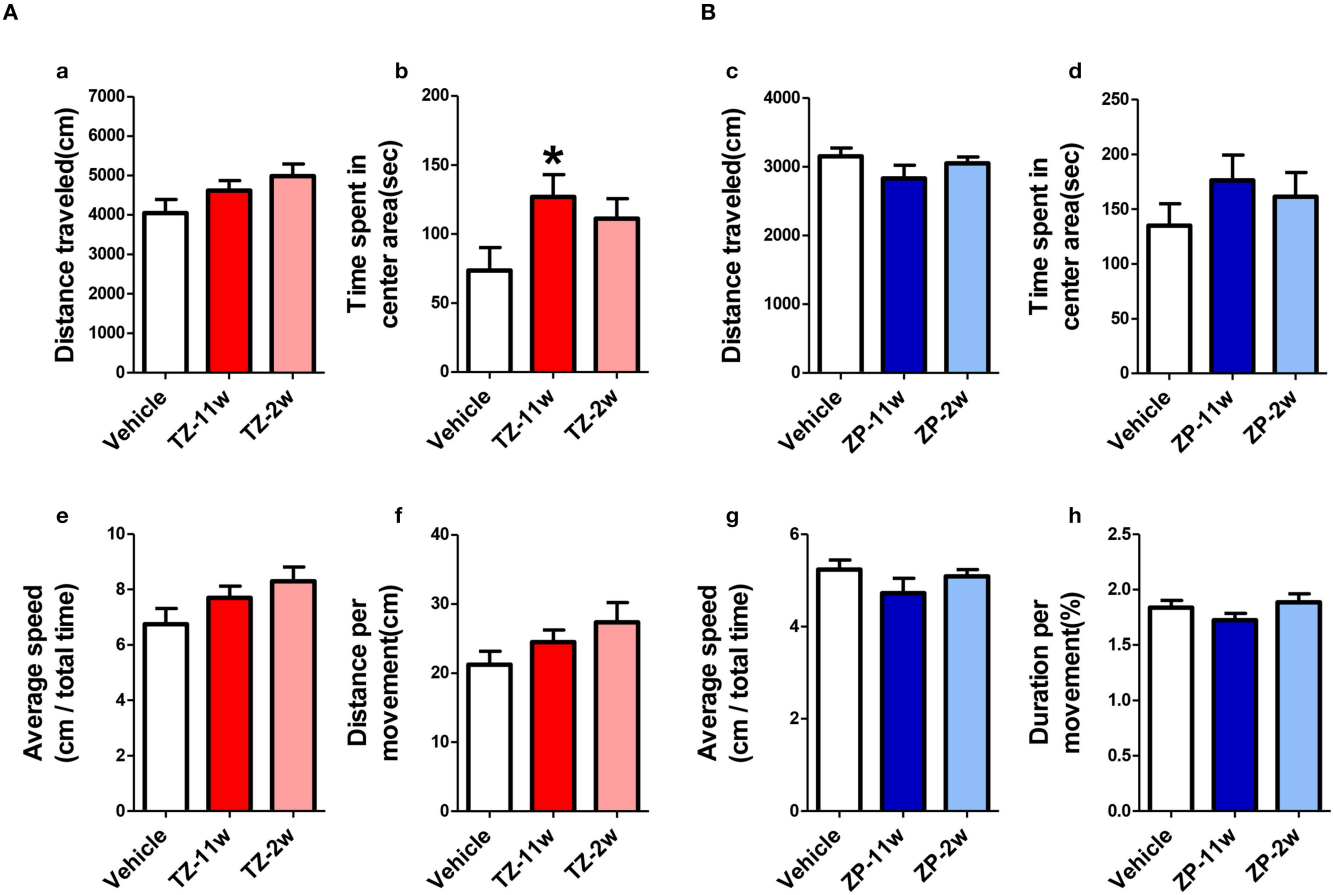
**Results of the open field (OF) test. (A,B)** The scores of the OF test (total test time, 600 s) are shown. **(Aa,Bc)** Distance traveled (cm) during the test period (600 s) is shown. **(Ab,Bd)** Time spent in the center area (seconds) is shown. **(Ab)** Statistically significant increases were detected in the TZ-11w group. **(Ae,Bg)** Average speed (cm/total time) is shown. **(Af,Bh)** Distance per movement (cm) is shown. *n* = 8, mean ± SEM. Asterisk (^*^) indicates a statistically significant difference (^*^p < 0.05, Student's *t*-test) compared to the Vehicle group.

**Figure 3:**
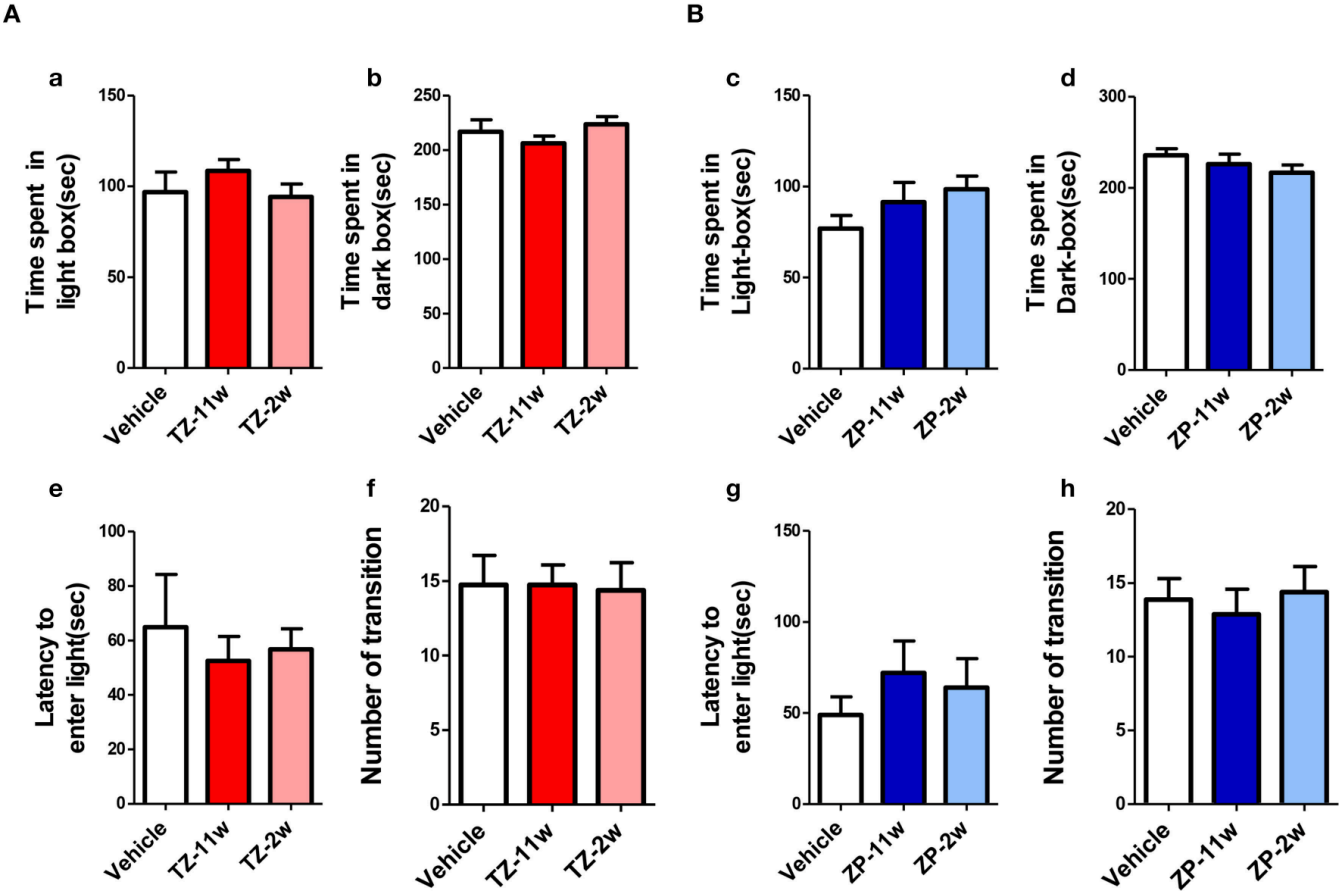
**Results of the light/dark transition (LD) test. (A,B)** The scores for the LD test (total test time, 360 s) are shown. **(Aa,Bc)** Time spent in the light box (seconds). **(Ab,Bd)** Light box distance traveled (seconds) is shown. **(Ae,Bg)** The number of transitions between the dark box and the light box. **(Af,Bh)** Latency (seconds) to enter the light box for the first time. No significant differences compared with the Vehicle group were detected in the LD. The number of mice per group was eight and the scores are shown as the mean ± SEM.

**Figure 4:**
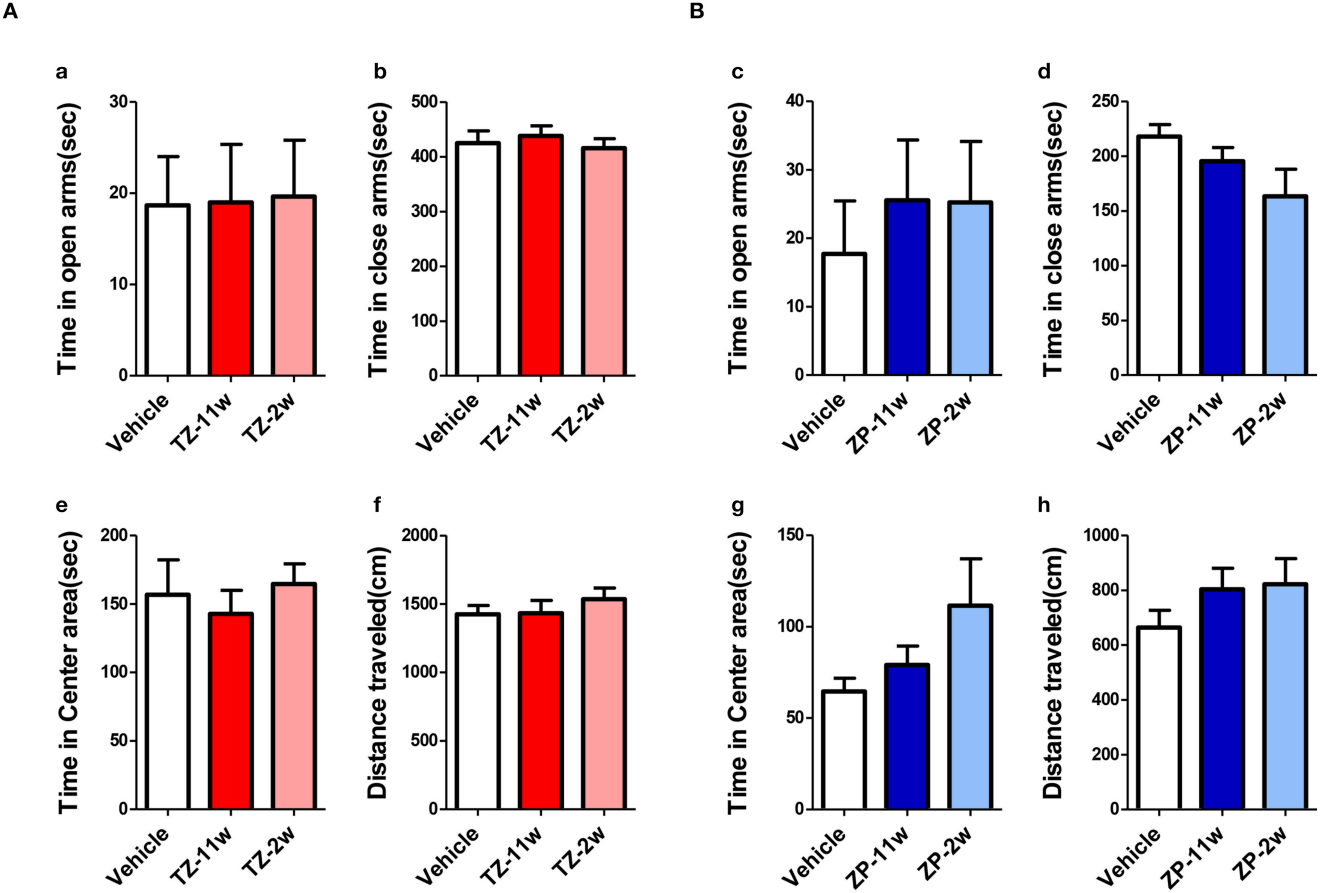
**Results of the elevated plus maze (EP) test**. EP tests were conducted to analyze emotional behavioral changes in TZ- and ZP-treated mice. **(A,B)** The scores of the EP test (total test time, 600 s) are shown. **(Aa,Bc)** Time spent in the open arm (seconds). **(Ab,Bd)** Time spent in the closed arm (seconds). **(Ae,Bg)** Time spent in the center area (seconds). **(Af,Bh)** Total distance moved (cm). No significant differences with the Vehicle group were detected in the EP. The number of mice per group was eight and the scores are shown as the mean ± SEM.

**Figure 5:**
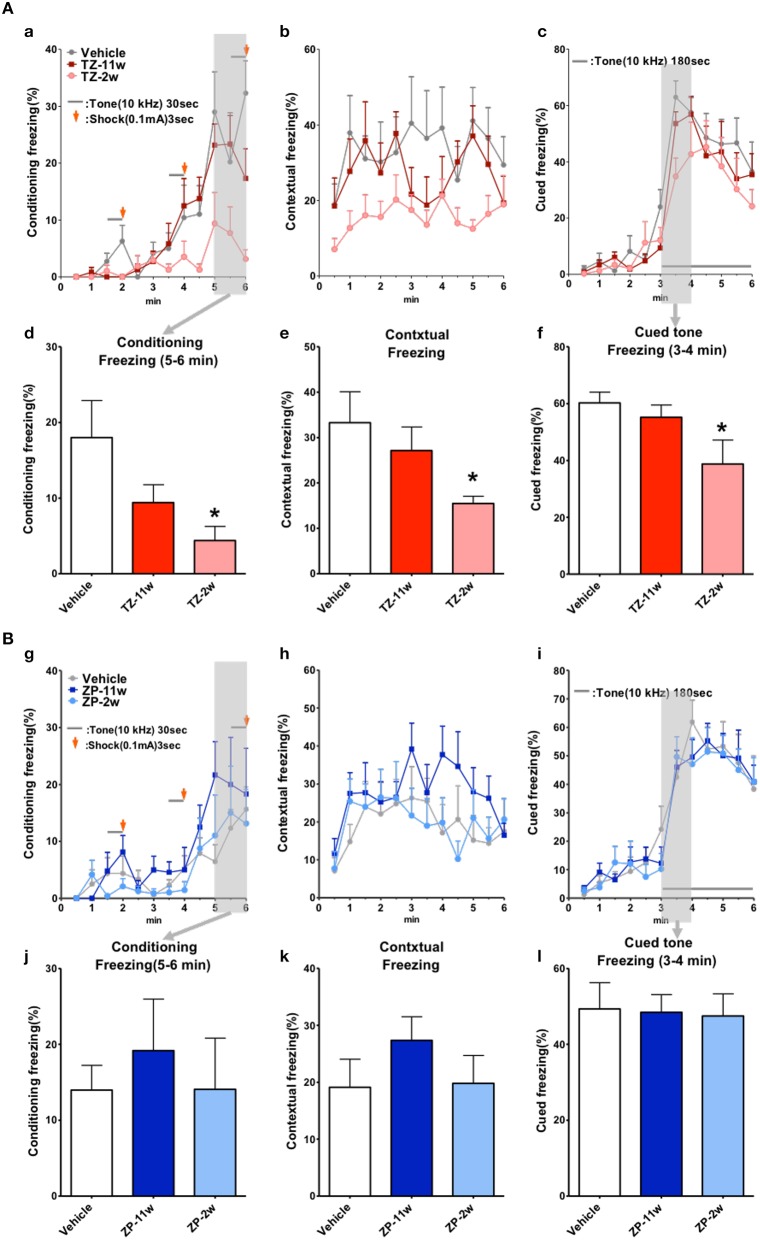
**Results of the fear conditioning test. (A,B)** Fear conditioning test was conducted to analyze the effects of TZ and ZP on learning and contextual memory, i.e., place (test-box) and sound (cued tone). **(Aa,d,Bg,j)** The total conditioning time was 360 s. Three cycles consisting of a tone (30 s) and a mild foot shock (arrows: 0.1 mA, 3 s) were carried out for each mouse after it was allowed to explore the box freely for 90 s. **(Aa,Bg)** The time course of the freezing % scores is plotted for the conditioning test. As the cycles of the conditioning were repeated, the freezing percentages increased in the Vehicle group and in all groups other than the TZ-2w group. This indicates successful conditioning. On the other hand, the freezing % remained low in the TZ-2w group. **(Ad,Bj)** The average freezing % scores during the later period (180–360 s) of the conditioning test are shown. A significant decrease in freezing % was observed in the TZ-2w group. **(Ab,e,Bh,k)** Contextual tests were conducted 24 h after the conditioning test to analyze the effects of triazolam on place memory function. **(Ab,Bh)** The time course of the freezing % scores is plotted for the contextual test. The total time of the test is 360 s. **(Ae,Bk)** The average freezing % scores in the contextual test are shown. A significant decrease in freezing % was detected in the TZ-2w group. **(Ac,f,Bi,l)** The cued test was conducted 24 h after the contextual test to analyze the effects of triazolam on cued memory function. The total time of the test is 360 s. **(Ac,Bi)** The time course of the freezing % scores was plotted for the cued test. The tone was presented to the mice during the later period of the test (180–360 s). **(Af,Bl)** The average freezing % scores for the first one minute period after the tone are presented. The freezing % scores of the TZ-2w group were significantly lower than those of the Vehicle group. The number of mice per group was eight and the scores are shown as the mean ± SEM. Asterisk (^*^) indicate statistically significant differences (^*^p < 0.05, Student's *t*-test) compared to the Vehicle group.

**Figure 6:**
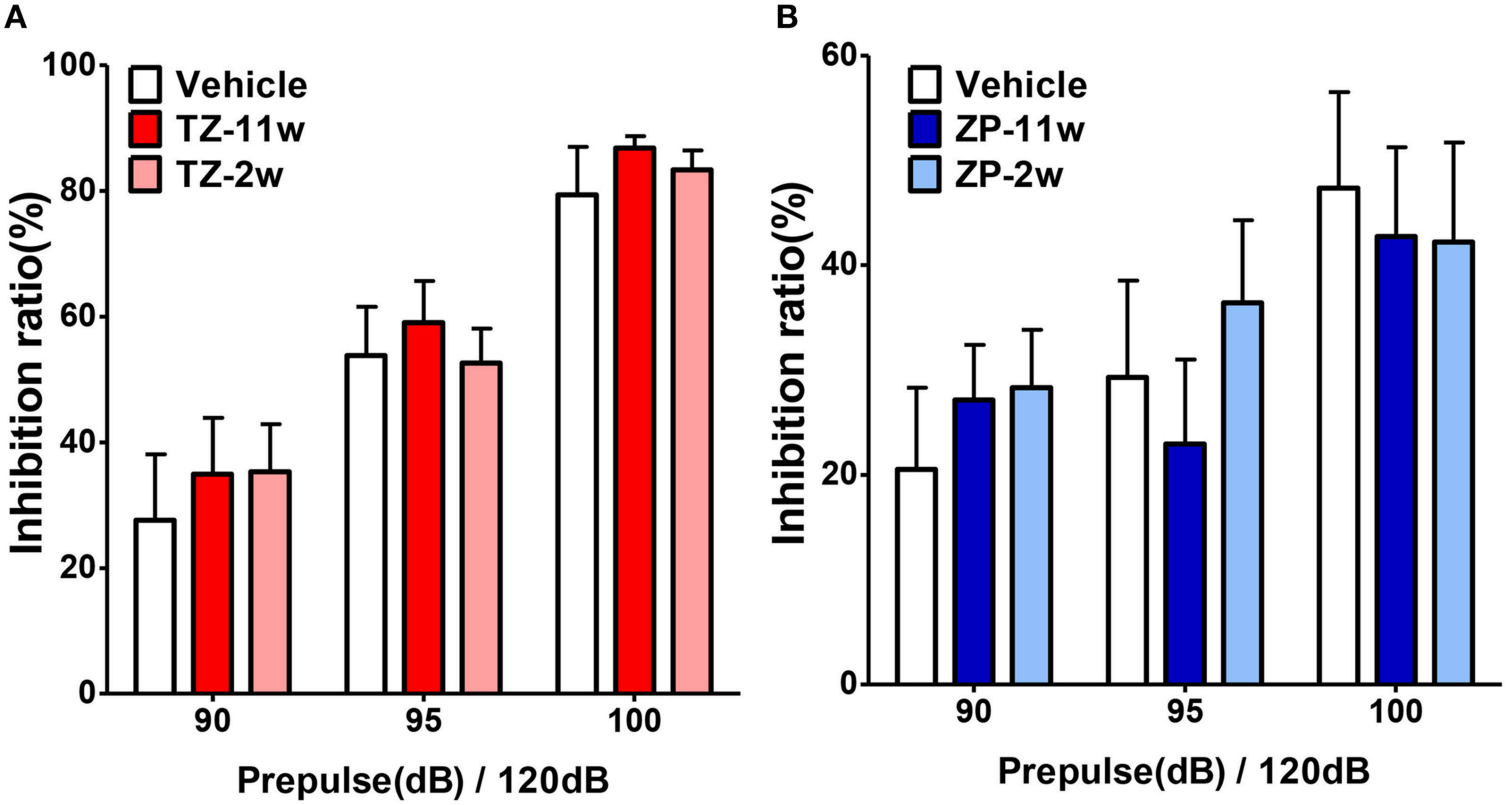
**Results of the pre-pulse inhibition (PPI) test. (A,B)** The pre-pulse inhibition test was conducted to analyze the effects of sleep-inducing drugs on sensory information processing. The scores indicated the inhibition (%) of the startle response to a 120-dB sound with pre-pulse sounds of 90, 95, 100, and 105 dB when compared to the response to a 120-dB sound without a pre-pulse sound. No significant differences were found in the PPI test. The number of mice per group was eight and the scores are shown as the mean ± SEM.

In the open field test (Figure 2), the total distance traveled and the time spent in the center region were significantly longer in the TZ-11w group (Figure 2Ab). Exposure to either chemical did not lead to any changes in the results of the light/dark transition test (Figure 3) or the elevated plus maze test (Figure 4). In the fear conditioning test, only the TZ-2w group did not show an increase in freezing % during the later period of the conditioning trial (Figures 5Aa,d, Bg,j, *p* < 0.05). In addition, in the contextual test, only the TZ-2w group had significantly lower freezing percentages (Figures 5Ab,e, Bh,k, *p* < 0.05). All other groups responded quickly to the tone and showed high freezing responses, while mice in the TZ-2w group had slower responses to the tone, which resulted in lower freezing responses (Figures 5Ac,f, Bi,l, *p* < 0.05). These results indicate that the administration of TZ during the juvenile period induces deficits in learning and memory. Exposure to either chemical did not lead to any changes in the results of the pre-pulse inhibition test (Figure 6). Significant differences were not observed in any of the tests in our BBT in the ZP-11w and ZP-2w groups. The results of the series of BBT performed on the TZ-11w, TZ-2w, ZP-11w, and ZP-2w groups during the adult stage are summarized in Table 1.

**Table 1:** **A summary for the behavioral change at adult stage with different developmental exposure of triazolam 1 mg/kg B.W. and zolpidem 50 mg/kg B.W**.

**Test name**	**Behavioral tasks**	**Triazolam 1 mg/kg**		**Zolpidem 50 mg/kg**	
		**TZ-11w**	**TZ-2w**	**ZP-11w**	**ZP-2w**
Open field	Distance traveled	1.14	1.23	0.90	0.97
	Center region time	1.72^*^	1.51	1.31	1.20
	Average speed	1.14	1.23	0.90	0.97
	Moving speed	1.03	1.09	0.96	0.97
	Moving episode number	0.99	0.98	1.00	0.97
	Total movement duration	1.12^*^	1.14^*^	0.93	1.00
	Distance per movement	1.15	1.29	0.91	1.00
	Duration per movement	1.14	1.19	0.94	1.03
Light/Dark transition	Dark distance	0.95	0.96	0.86	0.89
	Light distance	1.08	0.92	1.06	1.18
	Dark time	0.95	1.03	0.96	0.92
	Light time	1.12	0.97	1.19	1.28
	Number of transitions	1.00	0.97	0.93	1.04
	Latency to enter light	0.81	0.87	1.47	1.31
Elevated plus maze	Total distance	1.01	1.08	1.21	1.24
	Total center time	0.91	1.05	1.22	1.73
	Total open area	1.02	1.05	1.45	1.43
	Total close area	1.03	0.98	0.90	0.75
	Total arm select number	0.90	0.97	1.41	1.47
Fear conditioning	Total conditioning freezing %	0.83	0.27	1.57	0.91
	Conditioning 5–6 min freezing %	0.52	0.24^*^	1.39	1.02
	Total contextual freezing %	0.82	0.47^*^	1.43	0.72
	Cued tone 3–6 min freezing %	0.89	0.72	0.98	0.96
	Cued tone 3–4 min freezing %	0.92	0.64^*^	0.92	0.93
Pre-pulse inhibition	Pre-pulse 90 dB	1.07	1.28	1.32	1.38
	Pre-pulse 95 dB	1.19	0.98	0.78	1.24
	Pre-pulse 100 dB	1.07	1.05	0.90	0.89

The number indicates the fold change to vehicle and the asterisk indicates for the tests with the statistically significant difference to vehicle group [

* *p < 0.05 (Student's t -test)]*.

### Protein expression in the adult hippocampus in the TZ-11w, TZ-2w, ZP-11w, and ZP-2w groups

**Figure 7:**
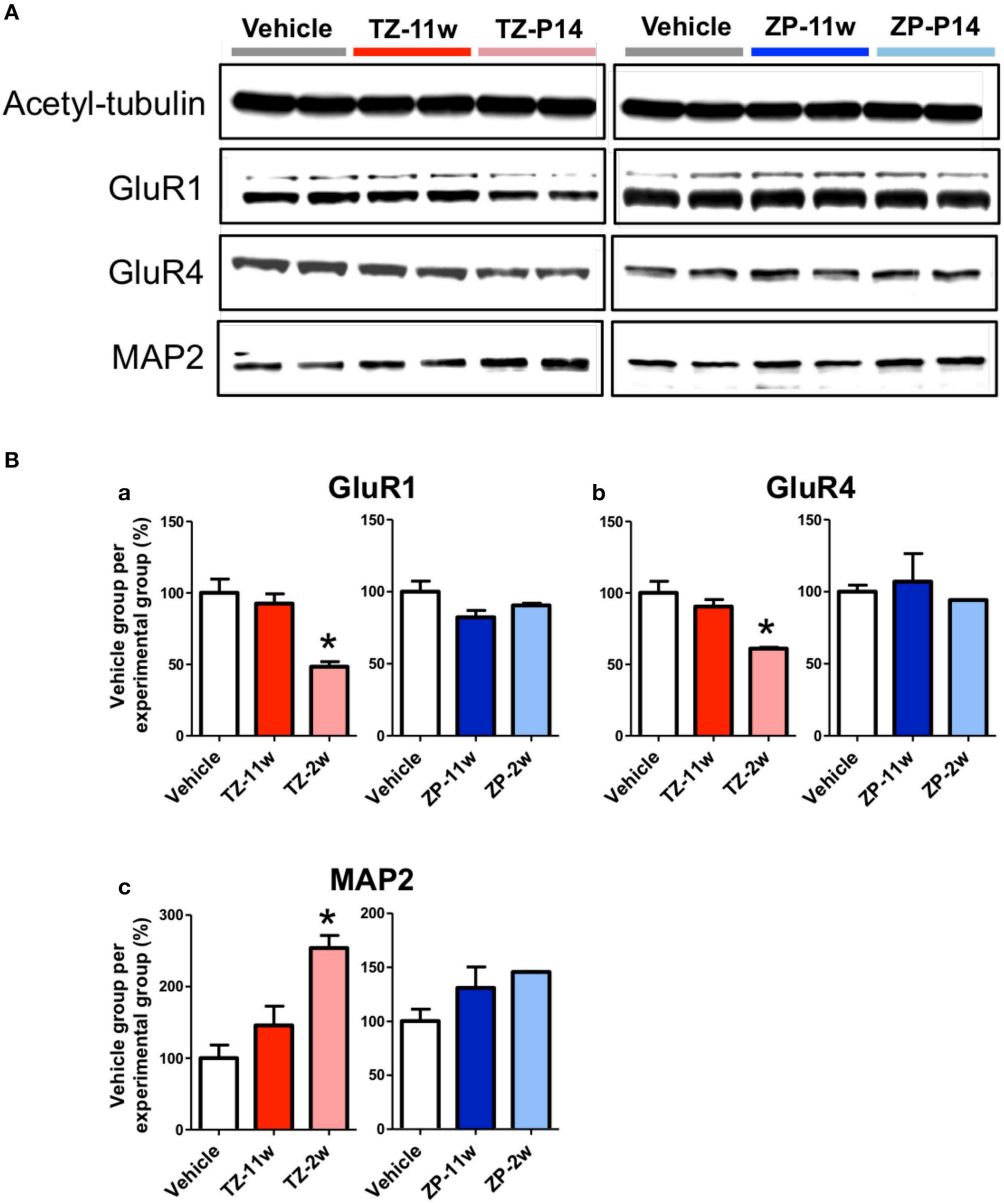
**Protein expression in the hippocampus during the adult stage following exposure to TZ and ZP during development**. The expression levels of several proteins were analyzed by western blotting to explore the effects of triazolam on the hippocampus during adulthood. **(A)** Acetyl-tubulin and AMPA-type glutamate receptors GluR1 and GluR4, as well as the neuronal cytoskeletal marker MAP2. **(Ba–c)** The expression of each protein was normalized to that of acetyl-tubulin expression and its ratio (%) to the expression in the Vehicle group is shown. The number of samples was two and the value is shown as the mean ± SEM. Asterisk (^*^) indicate statistically significant differences (^*^p < 0.05, Student's *t*-test) compared to the Vehicle group.

We analyzed expression of several proteins in the adult mice hippocampus following juvenile or adult stage exposure to triazolam or zolpidem using western blotting (Figure 7). Although, no differences were detected in the TZ-11w, ZP-2w, and ZP-11w groups, MAP2 (Figures 7A,Bb, *p* < 0.05) was increased and GluR1 and GluR4 (Figures 7A,Ba,b, *p* < 0.05) were decreased in the TZ-2w group. Both GluR1 and GluR4 belong to the AMPA-type glutamate receptor family and are known to be necessary for long-term potentiation in the hippocampus (Sanderson et al., 2008; Lee and Kirkwood, 2011). Therefore, decreases in the levels of GluR1 and GluR4 may be important mechanisms underlying the learning and memory deficits in mice exposed during the juvenile period.

### mRNA expression of immediate early response genes (IEGs) in the TZ-2w and ZP-2w groups

**Figure 8:**
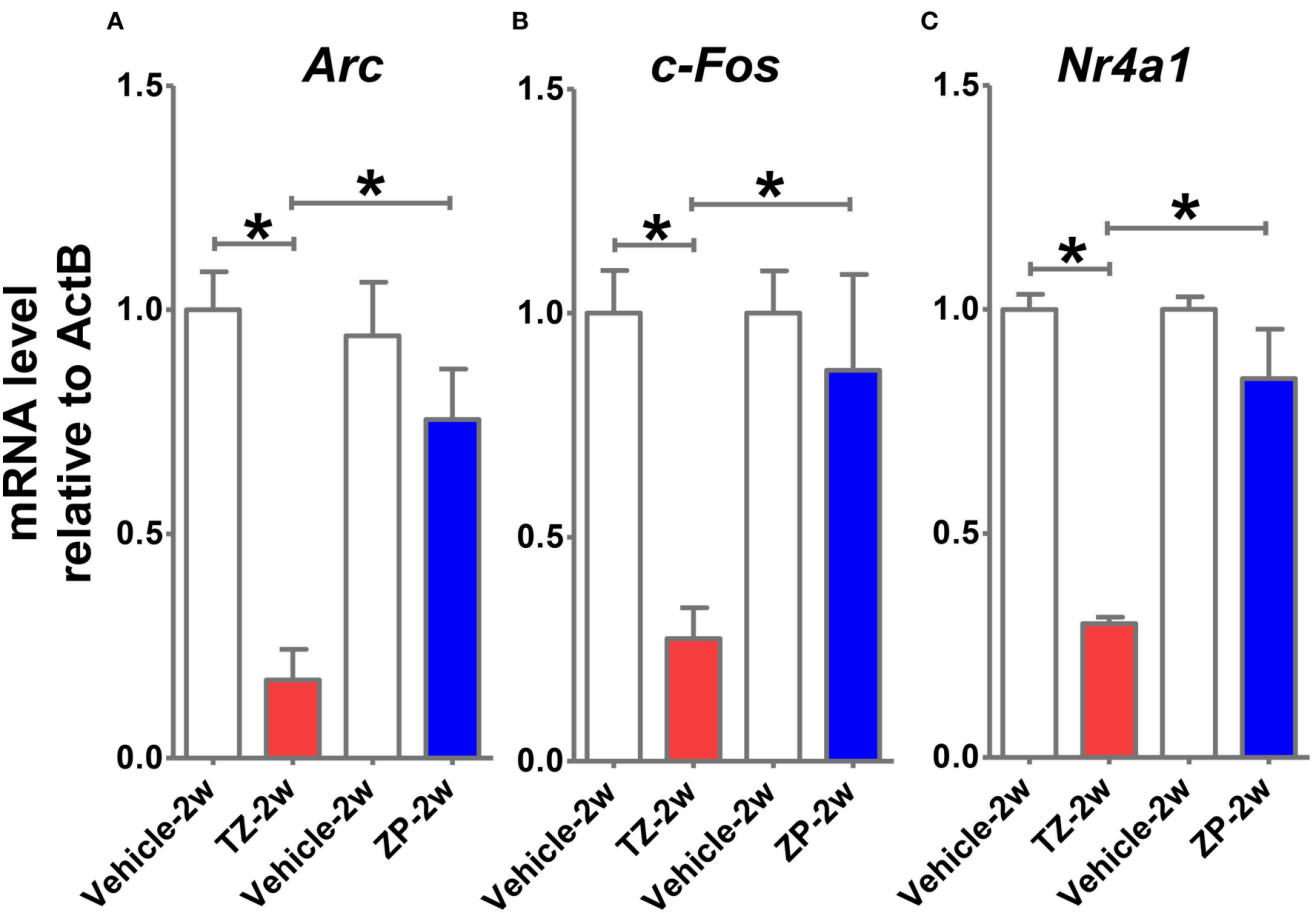
**Comparisons of immediate early response gene (IEG) mRNA expression levels in the hippocampus shortly after TZ or ZP exposure**. Eight hours after TZ or ZP administration to juvenile (2-week-old) mice, we examined the mRNA expression levels of multiple IEGs in the hippocampus. Significant decreases in the expressions of **(A)** *Arc*, **(B)** *c-Fos*, and **(C)** *Nr4a1* were observed in the TZ-2w group. The number of the samples in each group was three and the values are shown as mean ± SEM. Asterisk (^*^) indicate statistically significant differences (^*^p < 0.05, Student's *t*-test) compared to the Vehicle group.

We analyzed mRNA expression levels of IEGs (*Arc, c-fos*, and *Nr4a1*) in the hippocampus shortly (8 h) after TZ and ZP administration during the juvenile period. We observed decreased mRNA expression of IEGs in the TZ-2w group, but not in the ZP-2w group (Figure 8).

## Discussion

In this study, we analyzed the late-onset effects of the stimulation of the GABA-R signal using a behavioral battery tests (BBT) and several biochemical assays. We stimulated the GABA-R signal of mice by the oral administration of the sleep-inducing drugs TZ (1 mg/kg body weight [B.W.]) or ZP (50 mg/kg B.W.) during juvenile (TZ-2w, ZP-2w) and adult stages (TZ-11w, ZP-11w).

The results of the BBT indicate that spontaneous activity, as measured in the open field test, was significantly increased in the TZ-11w group. Reactivity to a novel environment may be changed in mice exposed to TZ during adulthood. On the other hand, deficits in learning and memory were detected by the fear conditioning test only in the TZ-2w group. The freezing response was decreased in all three stages (conditioning test, contextual test, and cued test) of the fear conditioning test. Although, the TZ-11w, ZP-11w, and ZP-2w groups had increases in freezing % as the cycles of tone and mild foot-shock were repeated, the TZ-2w group did not have an increase in freezing %, even during the later period of the test. These results may indicate that TZ-2w mice lose their ability to make short-term memories, which are needed for quick responses in this situation. In addition, TZ-2w mice had significantly lower scores in the contextual test. This may indicate deficits in spatial memory (Clark and Squire, 1998). In the cued test, a delay of the freezing response to the tone was detected in the TZ-2w group, which may indicate mild deficits in cued memory. We believe that these learning and memory deficits are the most serious effects of TZ treatment in the TZ-2w group.

To identify the molecular mechanisms underlying the late-onset learning and memory deficits, we first analyzed the expressions of several proteins in the hippocampus. We observed late-onset protein expression changes in the TZ-2w group. For example, MAP2 expression was increased in TZ-2w mice. MAP2 is a protein specifically expressed in the dendrites of neurons and plays an important role in the stabilization of tubulin structure in neuronal filaments (Caceres et al., 1983). There are also several reports of decreases in MAP2 levels in Alzheimer's disease and Parkinson's disease (Li et al., 2008; Liu et al., 2011). However, no increases in MAP2 levels have been reported thus far. It is therefore still unclear how this increase in MAP2 levels relates to the deficits induced by triazolam. In addition, the expression levels of GluR1 and GluR4 were decreased in the TZ-2w group. GluR1 and GluR4 are AMPA-type glutamate receptors and are known to be important for learning and memory, as they induce long-term potentiation and synaptic plasticity (Sanderson et al., 2008; Lee and Kirkwood, 2011). Therefore, the deficits of learning and memory in the adult stage in neonatally exposed mice may be related to decreases in GluR1 and GluR4 levels. None of the ZP-treated groups and TZ-11w groups had changes in MAP2, GluR1, or GluR4 protein expression.

We hypothesize that TZ may have greater inhibitory effects on the hippocampus during the juvenile period and that may be a trigger of learning and memory deficits. Therefore, we measured changes in IEG mRNA expression in the hippocampi of juvenile mice shortly after TZ or ZP exposure (8 h). As expected, TZ had a greater inhibitory effect on the juvenile hippocampus than ZP. Indeed, the expression levels of all three IEGs (*Arc, c-fos*, and *Nr4a1*) were decreased in response to TZ treatment. IEGs are known to be induced rapidly following neuronal activation and are considered as markers of activated neurons (Sheng and Greenberg, 1990). The TZ-specific suppression of neuronal activity during the juvenile period may thus be responsible for the TZ-specific learning and memory deficits.

In summary, our findings demonstrate that the BZD sleep-inducing drug TZ can lead to learning and memory deficits with juvenile exposure. In contrast, ZP, which is a non-BZD, did not induce deficits in brain functional development. Decreased IEG expression was detected in mice treated with TZ during the juvenile period, but not in mice treated with ZP during the juvenile period. Therefore, we suggest that decreased IEG expression may be one of the triggers for the long-lasting adverse effects of TZ on the brain. The learning and memory deficits induced by TZ may be dependent on the timing of the exposure, as GABA-R signal has different functions in different brain developmental stages and in different brain regions containing GABA-Rs (Rice and Barone, 2000). GABA-R signal is reported to have a critical role in the reconstruction of synapses during the juvenile period (Steward and Falk, 1986; Herschkowitz et al., 1997). Therefore, juvenile exposure to TZ may interfere with synapse reconstruction and affect the proper development of learning and memory.

We have previously reported the aberration of emotional behavior associated with deficits in learning and memory in adult male mice treated with domoic acid (the chemical compound for the excessive activation of glutamate receptor mediated signal; Glu-R signal) at prenatal period by the administration to pregnant female mice (Tanemura et al., 2009). Nevertheless, we could not find the correlativity with this study using TZ. The following points can be given as that reasons. In previous study, we used mice in prenatal period does not correspond in the degree of maturation of hippocampus from juvenile period (Rice and Barone, 2000; Luján et al., 2005). Moreover, the domoic acid and TZ are completely different mechanism of action. We demonstrated that TZ administration cause the IEGs expression inhibitory in the juvenile period hippocampus. However, the domoic acid administration is known to increase IEG expression in hippocampus (Scallet et al., 2004). For these reasons, current study cannot be directly compared with previous one. We guessed that there might be several differences between the effects induced by disturbances of GABA-R and Glu-R signals, as each neuronal signal has the particular function to construct the neuronal circuits and reorganization depending on brain development (Steward and Falk, 1986; Rice and Barone, 2000; Luján et al., 2005). In addition, we guessed that the various aberrations of behavioral manners might be induced by the various disturbances of neural signals by the neuroactive chemical compounds.

In conclusion, our study indicates that juvenile TZ exposure may lead to learning and memory deficits. GABA-R agonists, such as BZD, are used for the treatment of both sleep disorders and anxiety disorders in both adults and children (Chevreuil et al., 2010; Weiss and Garbutt, 2010; Pelayo and Yuen, 2012). Therefore, considering the possible influence of GABA-R agonists on brain development, their careful prescription to children is warranted.

## Author contributions

YF designed and performed experiments, analyzed data, and wrote the paper. KT designed the study, developed the methodology, conducted experiments, and editing the manuscript. KI helped with designed and editing the manuscript. MI helped with behavioral test and western blotting. KA, SK helped with the design of the study. MK, JK for the supervisor and design of the study.

### Conflict of interest statement

The authors declare that the research was conducted in the absence of any commercial or financial relationships that could be construed as a potential conflict of interest.
